# Current status and progress of research on the ADP-dependent glucokinase gene

**DOI:** 10.3389/fonc.2024.1358904

**Published:** 2024-03-25

**Authors:** Ningjing Guo, Qiong Luo, Qixian Zheng, Sheng Yang, Suyun Zhang

**Affiliations:** ^1^ Department of Oncology Medicine, Fujian Medical University Union Hospital, Fuzhou, Fujian, China; ^2^ Department of Respiratory Medicine, Fujian Medical University Union Hospital, Fuzhou, Fujian, China; ^3^ Department of Internal Medicine, Fujian Medical University Union Hospital, Fuzhou, Fujian, China

**Keywords:** ADP-dependent glucokinase, glycolysis, malignant tumours, targeted therapy, immunotherapy

## Abstract

ADP-dependent glucokinase (ADPGK) produces glucose-6-phosphate with adenosine diphosphate (ADP) as the phosphate group donor, in contrast to ATP-dependent hexokinases (HKs). Originally found in archaea, ADPGK is involved in glycolysis. However, its biological function in most eukaryotic organisms is still unclear, and the molecular mechanism of action requires further investigation. This paper provides a concise overview of ADPGK’s origin, biological function and clinical application. It aims to furnish scientific information for the diagnosis and treatment of human metabolic diseases, neurological disorders, and malignant tumours, and to suggest new strategies for the development of targeted drugs.

## 1 Introduction

Sugar is a type of carbohydrate, composed of carbon, hydrogen and oxygen, and is a vital nutrient needed by the human body, with its principal biological purpose being to supply energy and carbon for vital activities. The primary molecular form through which sugar is absorbed is monosaccharide. Glucose is one of the most commonly distributed and important monosaccharides in nature, providing the primary source of energy for living organisms.

Glucose catabolic pathways consist of anaerobic and aerobic oxidation. Cells take up glucose into the cytoplasm via the glucose transporter (GLUT) and convert it to pyruvate through glycolysis. In conditions of limited oxygen supply or utilization, pyruvate generation continues via lactate dehydrogenase (LDH) in the cytoplasm, resulting in lactate production. Conversely, under sufficient oxygen supply, pyruvate enters the mitochondria and undergoes aerobic oxidation. Anaerobic oxidation of 1 mole of glucose generates 2 moles of adenosine triphosphate (ATP), while complete oxidation generates CO2 and H2O, producing 30 or 32 moles of ATP. Therefore, the primary pathway for sugar catabolism to generate energy is through the aerobic oxidation of sugar. Additionally, the pentose phosphate pathway acts as a bypass originating from the glycolysis intermediate product glucose-6-phosphate. Although this pathway cannot produce ATP, it can provide ribose phosphate and reduced nicotinamide adenine dinucleotide phosphate (NADPH), which act as a carbon source and hydrogen donor for various anabolic metabolic processes within the body.

Glycolysis is a fundamental process in the catabolism of glucose that involves the breakdown of one glucose molecule into two pyruvate molecules. Hexokinases (HKs), which are metabolic enzymes, play a critical role in glycolysis by catalysing the initial step. This involves the phosphorylation of glucose to glucose-6-phosphate, which is an irreversible reaction that consumes ATP. Four isozymes of Hexokinases (HK I-IV) were identified in mammals quite early on. Irwin DM (1) utilised genomics to characterise hexokinase genes across a range of vertebrate species and close relatives, the investigation resulted in the discovery of a comparable gene, named hexokinase domain protein 1 (HKDC 1), which is now recognised as the fifth hexokinase.

ATP is generally regarded as the universal energy carrier for kinases as well as the most commonly used phosphate-based donor. However, the ADP kinase family, which phosphorylates sugars with ADP rather than ATP, was initially discovered in archaea and includes ADP-dependent enzymes such as glucokinase (ADP-GK), phosphofructokinase (ADP-PFK), and the bifunctional ADP-dependent glucokinase/phosphofructokinase (ADP-GK/PFK) (2–4). The dependency on ADP might be due to its ability to activate sugars during low energy reserves, specifically after a period of starvation, where ATP levels may decrease significantly, whilst relatively high ADP levels could still phosphorylate the sugars.

Early studies in tumour biology have recognized that alterations in glucose metabolism are a significant feature that distinguishes tumour tissues from normal tissues. Nearly 100 years ago, the German scientist Otto Warburg observed that tumour cells predominantly employ glycolysis to generate energy, even when oxygen is present. This metabolism, known as the Warburg effect, is characterised by high glucose consumption, low ATP synthesis and high lactate acid generation (5). It is possible that ADPGK plays a role in the Warburg effect.

ADPGK possesses a distinctive molecular mechanism in glucose metabolism and holds significant promise for diagnosing and treating an array of human diseases. This paper concisely summarises the present-day research status and progress in the origin, biological function and clinical application of ADPGK.

## 2 Overview of ADPGK

### 2.1 Origin of ADPGK

In 1994, the pattern of glucose metabolism of hyperthermophilic archaeon Pyrococcus furiosus was studied by Serve W. M. Kengen et al. (2) The study showed that the Embden-Meyerhof pathway was the main pathway of glucose catabolism and identified a new type of glucokinase, ADP-dependent glucokinase, unlike the typical ATP-dependent glucokinase. Subsequent sequencing of mouse and human genomes uncovered limited but evident homology to archaea. Mammalian ADPGK was discovered in 2004 due to its sequence similarity to archaeal ADPGK by Ron S. Ronimus et al. (6) The study involved cloning the recombinant ADPGK gene from an intact cDNA of Mus musculus. The gene responsible for encoding mouse ADPGK is found on chromosome 9. The research demonstrates that this newly discovered mouse enzyme is capable of using ADP as a phosphogroup donor to catalyse glucose phosphorylation. ADPGK was found to demonstrate specificity towards glucose through characterization. It displayed a lower phosphorylation activity towards mannose and fructose and exhibited a high affinity for glucose and ADP. Additionally, its activity was inhibited by high concentrations of glucose and its product, AMP. In contrast to hexokinase, ADPGK was not inhibited by the product glucose-6-phosphate. In 2012, Susan Richter et al. (7) demonstrated through their research that recombinant human ADPGK utilized ADP instead of ATP to phosphorylate glucose.

### 2.2 Biological functions of ADPGK

The ADPGK gene, located on chromosome 15q24.1 in humans, is a 54 kDa monomeric protein. High expression levels of ADPGK have been observed in many normal tissues with a preference for hematopoietic lineage cells including macrophages, monocytes, dendritic cells, T cells and B cells (8, 9). Additionally, the database indicates high expression of ADPGK in tumours and differential expression in comparison to normal tissues (7, 10).

ADPGK exhibits atypical features as it utilizes ADP instead of ATP as a phosphoryl donor, thus facilitating the phosphorylation of glucose to glucose-6-phosphate.Unlike hexokinase, ADPGK is not inhibited by the end product glucose-6-phosphate, can take up large amounts of glucose, and its expression is not regulated by hypoxia or hypoxia-inducible factor-1, so it is clear that the main advantage of ADPGK is to conserve ATP during ischaemia and hypoxia by reducing the initiation cost of glucose phosphorylation (7).

Tumour cells typically exhibit the Warburg effect and display high intrinsic production of reactive oxygen species (ROS) along with constitutive activation of the NF-kB pathway (11–13). In a study conducted by Marcin M. Kaminski et al. (14) in 2012, it was identified that T cell activation is driven by ADPGK linking enhanced glycolysis with mitochondrial reactive oxygen species generation. Mitochondrial production of reactive oxygen species (ROS) and expression of genes driven by nuclear factor kappa B (NF-kB) during T cell activation rely on ADPGK activation. The latter leads to an increase in glycolysis metabolism, through T cell receptor (TCR)-induced glucose uptake. The downregulation of mitochondrial respiration and glycolysis in favour of the mitochondrial glycerol-3-phosphate dehydrogenase 2 (GPD) shuttle suggests a transition to aerobic glycolysis, reminiscent of the Warburg effect. Additionally, the study identified ER localization of ADPGK and its protruding active site into the cytoplasm. The interaction between signaling and metabolic pathways that result in the release of mitochondrial ROS may have implications for tumourigenesis. Although ADPGK plays a critical role in remodeling energy metabolism induced by TCR activation, the exact mechanism remains unclear. Studies have shown that O-GlcNAc modification or complex N- and O-glycosylation are essential for activating T cells (15, 16). In 2019, researchers led by Roland Imle (17) confirmed the localization of ADPGK within the endoplasmic reticulum, and illustrate that ADPGK is part of a glucose sensing system in the ER modulating metabolism via regulation of N- and O-glycosylation, ADPGK deficiency resulted in reduced glucose uptake, diminished activities of hexokinase, phosphofructokinase, and respiratory chain complexes, activation-induced depletion of thymidine metabolism intermediates, and enhanced activation of autophagy, ultimately leading to a severe energy metabolism disorder.

ADPGK plays a vital part in the glycolytic process of archaea, but its function in most eukaryotic cells remains unknown. Overexpression of ADPGK in H460 and HCT116 human tumour cells did not lead to cell proliferation or glycolysis, and inhibition of ADPGK by siRNA did not result in the inhibition of glycolysis or cell death due to hypoxia (7). This indicates that although ADPGK is highly expressed in human tumour cell lines, it does not appear to contribute significantly to glycolysis. Further studies revealed that while oxygen consumption was generally reduced in ADPGK knockout cells, the knockdown of ADPGK in these cell lines had no discernible impact on glycolysis or extracellular acidification. Additionally, under normal growth conditions, knocking down ADPGK had no effect on cell survival. However, ADPGK protects H460 cells under stress conditions of hypoxia and reduced glycolysis (such as silencing of HK2), but this protection is not suitable for HCT116 cells (18). It should also be noted that ADPGK can enhance tumour cell survival under highly glycolysis-dependent conditions. Nevertheless, the phenotype elicited by ADPGK knockout varies depending on the cell line. Therefore, further comprehensive investigation is needed to unravel the specific biological function of ADPGK.

## 3 Clinical application of ADPGK

### 3.1 ADPGK and metabolism related research

Energy metabolism is a fundamental feature of the body’s vital processes, while cell growth and proliferation necessitate energy support. ATP provides cells with energy, which is mainly generated through glucose catabolism. ADPGK, a glycolytic enzyme, participates in glucose metabolism process, which aids in the maintenance of the body’s material and energy homeostasis.

In 2006, Amanda H. McDaniel et al. (19) investigated the gene responsible for variation in fat levels in chromosome 9 of mice and determined that ADPGK, a gene with functional importance for body fat phenotype, plays a metabolic role. In 2018, in order to further identify genes involved in human BMI loci, Thomas J. Baranski (20) conducted a high-throughput screen in Drosophila melanogaster and found that ADPGK was associated with human BMI single nucleotide polymorphism. In 2020, Pankaj Kumar et al. (21) found that mutations in ADPGK, a BMI-associated target, can disrupt the binding activity of miRNAs to their targets. It seems that ADPGK exerts some influence on obesity through its role in carbohydrate metabolism.

In 2020, Jing Pan (22) conducted a proteomics study on fetal membranes. The study revealed that the protein expression of ADPGK and PKM related with glycolytic metabolism was significantly different between the preterm and term groups. The study’s data revealed heightened ADPGK levels, which is the rate-limiting enzyme in glycolysis’s initial stage, and reduced PKM levels, the enzyme that catalyses glycolysis’s ultimate step, in the preterm delivery group. This indicates that an imbalance in glycolysis can reduce energy synthesis in fetal membrane tissue, leading to preterm birth.

In 2021, Rongyu Yao et al. (23) discovered single nucleotide polymorphisms (SNP) in the 3′-UTR of the ADPGK gene are strongly associated with reproductive organ parameters and sperm counts in the Hu sheep. The energy supplied by glycolysis promotes testicular development and spermatogenesis through the action of ADPGK. Hu sheep with high levels of ADPGK gene expression showed notable benefits in reproductive performance. Thus, the polymorphism of this gene could serve as a promising DNA marker in the Hu sheep breeding programme.

Skeletal muscle plays a crucial role in both exercise and metabolism, and its growth, development, and injury repair directly impact the body’s ability to exercise. Regular exercise training can enhance the enzyme activity responsible for glucose metabolism in skeletal muscle, in contrast, overtraining may inhibit the relevant enzyme activity, thereby impeding glucose metabolism (24). In 2022, Ethan Moreland et al. (25) demonstrated through further analyses based on polygenic mapping of elite strength athletes that the likelihood of becoming a good strength athlete depends on the carriage of a large number of strength-related alleles, including the ADPGK gene, which is associated with glucose metabolism.

### 3.2 ADPGK and nervous system related research

In 2021, Kai Zhang et al. (26) created a mouse model of sciatic nerve injury and conducted proteomics studies, revealing a significant increase in the protein level of ADPGK in the injured sciatic nerve. Further research identified that ADPGK was specifically expressed and up-regulated in macrophages, with injured axons capable of independently promoting the upregulation of ADPGK in macrophages. Additionally, overexpression of ADPGK led to lipopolysaccharide-induced macrophage activation. This study highlights the potential role that ADPGK seems to play in enhancing injury-induced macrophagocytosis by macrophages *in vivo*, making it a promising target for future therapies in neurological diseases.

### 3.3 ADPGK and tumour related research

One important characteristic of tumour cells is their abnormal energy metabolism. Normal cells primarily depend on mitochondrial oxidative phosphorylation to metabolise glucose and produce energy. However, in low oxygen environments, glucose metabolism shifts to the glycolytic pathway. It is imperative to note that this phenomenon, commonly known as the Warburg effect, is a hallmark of cancer development. Tumour cells demonstrate high-speed glycolytic phenomena in various environments, including the Warburg effect (5), due to their requirement for malignant proliferation. This effect involves high glucose consumption, low ATP synthesis and high lactate production. According to biochemical analysis and biological model simulation, tumour cells that generate ATP using the “high rate-low yield” mode have a growth advantage when sharing energy sources with multiple cells (27). Glucose metabolism fulfils the cellular need for ATP and aids in preserving the intracellular redox equilibrium, while also supplying the carbon skeleton and NADPH required for the biosynthesis of biological macromolecules. Insufficient glucose supply indicates that tumour cells for glycolysis are more competitive than normal cells, providing a growth advantage for tumour cell proliferation (28). Moreover, lactate production increases, resulting in tumour microenvironment acidification that promotes several key carcinogenic processes, including angiogenesis, tissue invasion, metastasis and drug resistance (29).

Currently, ADPGK exhibits high expression in hormone-dependent breast and prostate tumours, promoting the uptake of glucose via glycolysis to stimulate tumour proliferation, migration, and invasion. Ji-Hyun Lee et al. (30) discovered *in vitro* that ADPGK driver mutations encouraged breast cancer cell migration, leading to alterations in EMT markers and facilitating metastasis in 2016. In 2019, Heba Alshaker et al. (31) discovered that the suppression of the oncogenic sphingosine kinase 2 (SK2) resulted in the decrease of ADPGK in cancer cells engaged in epithelial-mesenchymal transition (EMT), as highlighted by previous studies. Moreover, a 2021 study by Wang Fengxia et al. (32) indicated that gonadotropin inhibitory hormone (GnIH) in tumour cells can suppress ADPGK protein expression and induce apoptosis of MCF-7 cells in estrogen receptor-positive breast cancer. Hang Xu et al. (33) discovered in 2023 that ADPGK drives prostate cancer progression and high expression of it leads to poor prognosis in patients. ADPGK can accelerate prostate cancer glycolysis and progression through activation of ALDOC-AMPK signaling. This suggests that ADPGK could be an effective target and marker for prostate cancer treatment and prognosis assessment.

In 2020, Amol Tandon et al. employed ADPGK as a fresh glucose sensor and a probable tumour target for Burkitt’s lymphoma (BL) (10). It was discovered that the ADPGK knockout Ramos BL cells showed attenuated tumour aggressiveness *in vitro* and *in vivo*. ADPGK glycolysis is disrupted in knockout cells, resulting in a reduction of Warburg effector markers. This renders the cells unable to meet their heightened energy demands, thereby impairing their ability to undergo activation-induced differentiation. Further, it causes apoptosis and leads to a cumulative reduction in translocated proto-oncogene MYC mutations in knockouts.

So far, numerous molecular mechanisms concerning the Warburg effect in cancer exist, but they remain incompletely understood. Nonetheless, certain factors have been associated with this phenomenon, including the tumour microenvironment, signaling pathways that promote cancer, dysregulated metabolic enzyme expression, and others (34, 35). Numerous genetic and biochemical changes contributed to the molecular basis of the high rate of glycolysis in tumour cells, which included the elevated HK2 expression (36). The significant increase in HK2 expression in tumours is believed to result from metabolic reprogramming that meets the demands of accelerated tumour growth. Taking HK2 as the target of cancer therapy provides a new idea to break through the cancer problem. Similarly, whether the study of ADPGK is expected to provide new therapeutic measures for the development of targeted drugs?

Piotr Tokarz et al. (37) demonstrated the crystal structure of the archaeal ADPGK protein complexed with the pan-kinase inhibitor 5-iodotubercidin (5ITU), d-glucose, inorganic phosphate and a magnesium ion. Based on the fact that ADPGK can be involved in the regulation of T cell activation, i.e., TCR activation induces transient activation of ADPGK via the diacylglycerol (DAG) branch of the TCR signaling cascade, increased ADPGK activity in turn contributes to downstream mitochondrial ROS production and the ROS-dependent NF-κB transcriptional response (14), Piotr Tokarz et al. (37) further demonstrated that binding of 5ITU to the ADPGK active site inhibits reactive ROS release and downstream gene expression induced by human ADPGK-dependent T-cell activation, and can therefore be used as a model compound for further screening of human ADPGK-specific inhibitors. Moreover, 8-bromoadenosine phosphate (8-Br-AMP) is a competitive inhibitor of ADPGK and the bromine substitution leads to considerable changes in the structural activities of the protein site. It interacts with critical catalytic residues (38). This forms a pivotal foundation for the rational development of ADPGK inhibitors that can modulate immune responses and be utilized in immunometabolism research.

Tumour immunotherapy is an emerging therapeutic strategy which enables the patient’s immune system to fight against the cancer. Immune checkpoint inhibitors currently comprise one of the most effective immunotherapy approaches for cancer. The US Food and Drug Administration (FDA) has approved four immune checkpoints, including cytotoxic T-lymphocyte-associated protein-4 (Cytotoxic T-lymphocyte associated protein-4,CTLA-4), Programmed cell death-1 (PD-1), Programmed death ligand-1 (PD-L1), and Lymphocyte activation gene-3 (LAG-3) which are immune checkpoint inhibitors that have shown promising results in cancer treatment (39). Moreover, vaccine therapy aims to induce an immune response against specific tumour antigens, and research is focused on developing personalized therapeutic neoantigen vaccines. The immune system is stimulated by the tumour-specific antigens, which have been identified through gene sequencing technology, and designed based on the patient’s tumour characteristics (40, 41). However, most neoantigens exhibit low immunogenicity, a newly found bi-adjuvant neoantigen nanovaccine (banNV) addresses this issue. The vaccine administers a neoantigen peptide (Adpgk) from MC-38 colon cancer along with two adjuvants (Toll-like receptor (TLR) 7/8 agonist R848 and TLR 9 agonist CpG) to achieve potent cancer immunotherapy (42). In another study, a neoantigenic peptide (Adpgk) from MC-38 colon cancer was also supplemented with ten consecutive positively charged lysines (10 K-Adpgk) in order to obtain a cationic peptide, which self-assembled with the TLR-9 agonist, CpG oligodeoxynucleotide (CpG ODN) adjuvant, and directly formed antigen/adjuvant-integrated nano-partitioned complexes (PCNPs) via electrostatic interactions for potent tumour immunotherapy (43). The peptide from ADPGK shows potential as a tumour neoantigen in such immunotherapy. In addition, in 2020, Ying Jing et al. (44) employed a strategy that combined pharmacovigilance data and omics data to assess multiple omics factors and immune-related adverse events in various cancer types. The resulting report examines the association between the odds ratios and immune-related adverse events (irAEs). It has been demonstrated that Lymphocytoplasmic proteins 1 (LCP 1), a lymphocytic cytoplasmic protein, along with ADPGK, has the ability to predict irAEs accurately. This presents novel approaches to managing immunotherapy in patients with cancer.

## 4 Conclusion and outlook

**Figure 1:**
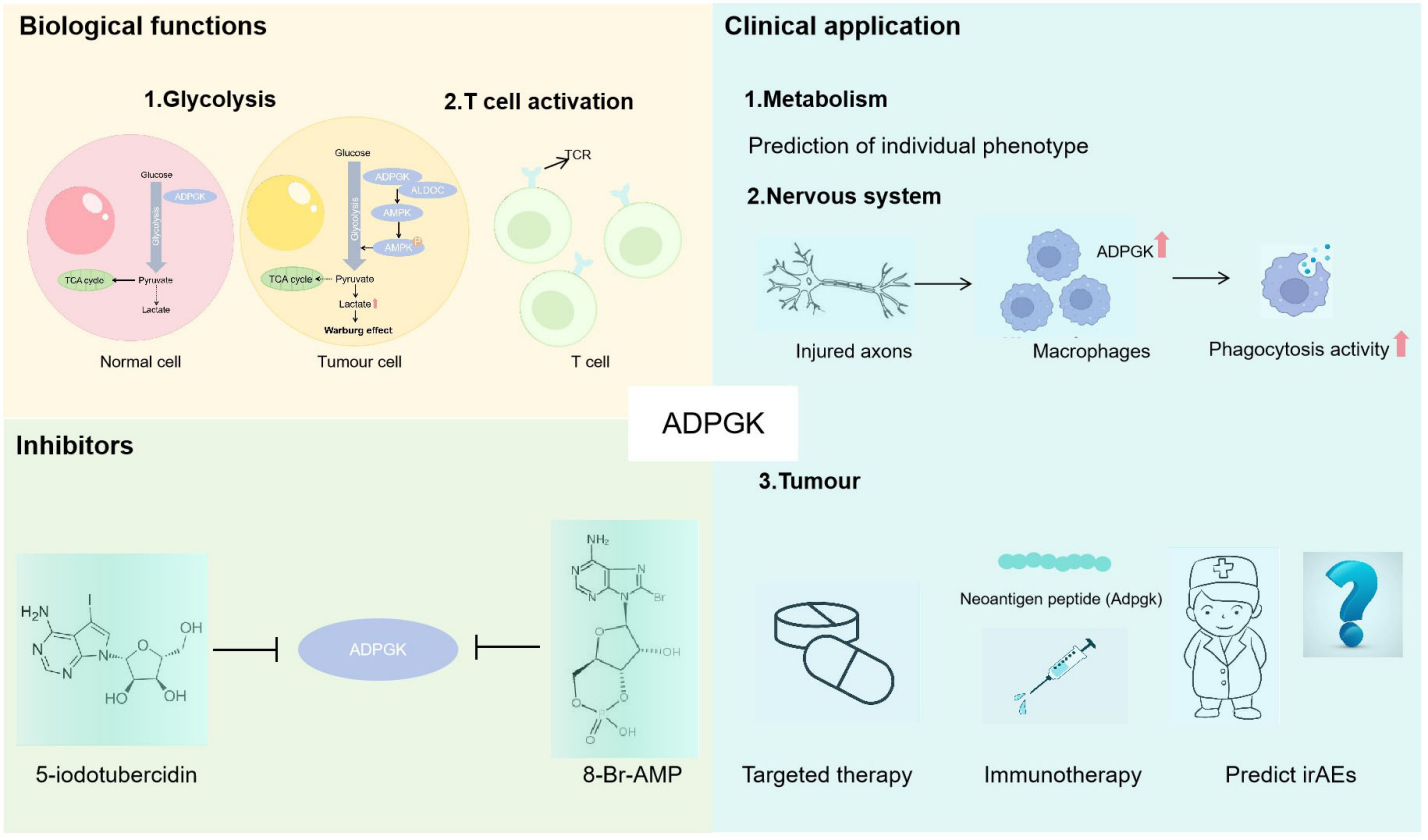
The role of ADPGK. Biological functions: 1. ADPGK is involved in glycolysis, ADPGK overexpression significantly promotes the process of glycolysis, and the overproduction of lactate induced by the Warburg effect further induces the cancer progression (33); 2. ADPGK can activate T cells. Clinical application: 1. ADPGK as a glycolytic enzyme can predict individual phenotype; 2. The injured axons can automatically promote the up-regulation of ADPGK in macrophages, and ADPGK overexpression promotes the phagocytosis activity; 3. ADPGK is expected to provide new ideas for targeted therapy and immunotherapy of tumours and predict immune-related adverse events. Inhibitors: ADPGK can be inhibited by 5-iodotubercidin and 8-Br-AMP.

In conclusion, ADPGK is capable of catalysing the glucose into glucose-6-phosphate, thereby playing a role in glycolysis. In contrast to hexokinase, ADPGK utilizes ADP as a phosphate donor and is unresponsive to inhibition by glucose-6-phosphate, hypoxia, and HIF-1. As a result, ADPGK exhibits increased activity during ischemia and hypoxia, enabling it to reduce the ATP consumption required for glucose phosphorylation, thus preserving energy homeostasis. The glycolytic function of ADPGK is thus vital for energy supply. Currently, the study demonstrates that ADPGK is linked to metabolic and nervous system diseases and has a close association with the development of malignant tumours. However, its biological functions, molecular mechanisms and clinical applications in eukaryotes need to be further studied and extensively explored. It is reasonable to believe that ADPGK could be used as a potential target to provide new strategies for the treatment of the disease (**Figure 1**).

## Author contributions

NG: Resources, Writing – original draft. QL: Resources, Writing – review & editing. QZ: Resources, Writing – review & editing. SY: Funding acquisition, Supervision, Writing – review & editing. SZ: Supervision, Writing – review & editing.
